# Selective and Colorimetric Detection of p-Nitrophenol Based on Inverse Opal Polymeric Photonic Crystals

**DOI:** 10.3390/polym12010083

**Published:** 2020-01-03

**Authors:** Lu Li, Tiantian Meng, Wanbin Zhang, Ying Su, Juan Wei, Xinwei Shi, Guanghua Zhang

**Affiliations:** 1 Key Laboratory of Auxiliary Chemistry and Technology for Chemical Industry, Ministry of Education, Shaanxi University of Science and Technology, Xi’an 710021, China; 2 The New Style Think Tank of Shaanxi Universities (Research Center for Auxiliary Chemistry and New Materials Development), Shaanxi University of Science and Technology, Xi’ an 710021, China; 3 Department of Chemistry, National University of Singapore, 3 Science Drive 3, Singapore 117543, Singapore; 4 Engineering Center of QinLing Mountains Natural Products, Shaanxi Academy of Sciences, Xi’an Botanical Garden of Shaanxi Province (Institute of Botany of Shaanxi Province), Xi’an 710061, China

**Keywords:** polymeric photonic crystals, structural color, p-nitrophenol

## Abstract

The detection of p-nitrophenol (PNP) is of great significance for assessment of environment pollution and potential health risks. In this study, based on inverse opal polymeric photonic crystals (IOPPCs), a selective and visual sensor for high-performance PNP detection is developed. Due to their unique optical properties, IOPPCs report events by change of color, which can easily be observed by the naked eye. Hydroxyethyl methacrylate (HEMA) was selected as the functional monomer with which to fabricate the IOPPCs. By precisely adjusting the molar ratio between the functional monomer and the crosslinker, the sensors were only able to be sensitive to a specific solution, thus realizing the visual, selective, and semi-quantitative detection of PNP. When the sensors were immersed in different concentrations of PNP solution, their Bragg diffraction wavelengths showed different redshifts. The color of the IOPPCs changed from green to red as the peak shift of Bragg diffraction occurred. In addition, the IOPPCs displayed good interference immunity and reusability.

## 1. Introduction

p-Nitrophenol (PNP), also known as 4-nitrophenol, is a typical phenolic compound. As a common industrial chemical, it is widely used in the manufacture of many kinds of raw materials, such as pesticides, pharmaceuticals, organic synthetic dyes, and pigments [1,2,3]. However, enormous usage of PNP is harmful to environment and especially to water, causing serious water pollution [2,3,4]. PNP has been found to be a highly toxic, carcinogenic, degradation-resistant and bioaccumulative compound which can the damage human nervous system, hemopoietic system, heart, liver, and kidneys, etc. It has also been listed as a priority pollutant for human beings and ecological environments [1,2,3,4]. Hence, the detection of PNP is of great significance for assessment of environmental pollution and potential health risks.

To date, conventional methods for the detection of PNP include spectrophotometry, atomic absorption spectrometry, mass spectrometry, ion chromatography, electrochemical devices, liquid (gas) chromatography–mass spectrometry (LC-MS and GC-MS), and so on, inevitably rely on large-scale analytical instruments [4,5,6,7,8]. Although these methods are mature and accurate, they require high cleanliness of samples and corresponding pre-treatments before detection, which is time-consuming and prevents realize real-time and fast on-site detection. Hence, developing a rapid and visual method for PNP detection is a challenging problem which must be overcome by excluding these disadvantages with rational design.

Photonic crystals, consisting of periodic arrangements of dielectric materials with different refractive indexes, produce color when the photonic stop bands are located in the visible range [9,10,11,12,13,14,15,16,17,18]. Because the response process of photonic crystals can be directly expressed by light diffraction signals (the structure color “naked eye” can be seen) without any marking technology or any other analytical instruments, much attention has been paid to it in the field of sensor design and application [19,20,21,22,23,24,25,26,27,28,29,30,31]. In a previous work we prepared a novel carbonate ion sensor for detection of carbonate ions by the naked eye based on inverse opal polymeric photonic crystals (IOPPCs) [32]. By precisely adjusting the molar ratio between the functional monomer and the crosslinker, the sensors are able to only be sensitive to a specific range of pH, thus realizing real-time, fast, and semi quantitative detection of carbonate ions. Inspired by this, we also successfully prepared a novel kind urea sensor with methacrylic acid as the functional monomer [33]. Meng’s group realized the detection of PNP through a combination of photonic crystals and molecular imprinting technology [34]. However, their sensor has many interference factors in polar solvents (especially in water) because the response mechanism of such polymeric photonic crystals based on molecular imprinting is mainly dependent on the intermolecular hydrogen bond, van der Waals force, and electrostatic attraction, etc. The whole process is also complicated.

In this work, based on IOPPCs, a selective and visual sensor for high-performance PNP detection is developed. Hydroxyethyl methacrylate (HEMA), a commercially available functional monomer, is used to build the IOPPCs. By precisely adjusting the molar ratio of the functional monomer and the crosslinker, the sensors are able to only be sensitive to a specific solution, thus realizing the visual, selective, and semi quantitative detection of PNP.

## 2. Materials and Methods

### 2.1. Materials and Chemicals

Methacrylic acid, acrylic acid, methyl methacrylate, acrylonitrile, 2-chloroacrylonitrile, 2-hydroxy-2-methylpropiophenone, hydroxyethyl methacrylate, ethylene glycol dimethylacrylate (EGDMA), and tetraethoxysilane (TEOS) were purchased from Alfa Aesar (Shanghai, China). Phenol, catechol, quinol, p-nitrophenol, resorcinol, pyrogallol, phloroglucinol, dipotassium hydrogen phosphate, lysine, 1,2,4-benzentril, sodium nitrite, sodium chloride, and potassium chloride were purchased from Aladdin (Shanghai, China). Ammonia, anhydrous ethanol, hydrogen peroxide (30% water solution), hydrofluoric acid, and sulfuric acid were all obtained from Beijing Chemical Reagent Ltd. Co (Beijing, China). Glass slides (25 mm long, 15 mm wide, and 1 mm thick) and polymethyl methacrylate (PMMA) slides (25 mm long, 15 mm wide, and 1 mm thick) were purchased from local suppliers (Laka, Xi’an, China). Before use, the glass slides were immersed in an H_2_SO_4_/H_2_O_2_ mixture (7:3, *v*/*v*) for 24 h and then repeatedly rinsed with deionized water in an ultrasonic bath. All small beakers for the fabrication of colloidal crystal templates were treated as well. The solvents and chemicals were of reagent grade and were used without further purification unless specially described.

### 2.2. Fabrication of Photonic Crystal Templates

SiO_2_ particles were synthesized by using an approach based on the Stöber method with certain modifications, as follows [33]. In a typical preparation process, anhydrous ethanol (100 mL), deionized water (6 mL), and ammonia (4 mL) were mixed in a 250 mL flask and stirred gently with a magnetic stirrer at 30 °C in a water bath. Then, 5 mL TEOS was quickly added and the reaction was terminated after about 8 h. The resultant particles were centrifuged and washed six times with ethanol. The resulting product was fully dispersed in anhydrous ethanol (0.5–1 wt %) and then added into small beakers. The glass slides were cleaned well by rinsing with ethanol and were placed vertically into each beaker for photonic crystal growth. After complete volatilization of the ethanol in a vacuum drying oven at 30 °C photonic crystal templates were obtained. An SEM image of the SiO_2_ particles is shown in Figure S1 (from the Supplementary Materials). The size of the SiO_2_ particles was about 202 nm and the polymer dispersity index (PDI) value was 0.017.

### 2.3. Preparation of Inverse Opal Polymeric Photonic Crystals

Typically, the prepolymer was made using HEMA (5 mM), 2-hydroxy-2-methylpropiophenone (10 μL), and EGDMA (1 mM), which were sufficiently mixed in anhydrous ethanol (1 mL). Then, the prepolymer was degassed with nitrogen for 5 min to remove the dissolved oxygen. The solution was infiltrated into the photonic crystal templates, which were covered with PMMA slides. Photopolymerization was performed under a UV light at 365 nm for 3 h. Inverse opal polymeric photonic crystals were obtained after the films were immersed in 1% HF aqueous solution and then washed to neutral with deionized water and immersed in deionized water to reach equilibrium swelling. An SEM image of the IOPPCs is shown in Figure S2 (from the Supplementary Materials) and schematic illustrations of the preparation of the IOPPCs are shown in Figure S3 (from the Supplementary Materials).

### 2.4. Characterization

The morphology and microstructure of the colloidal particles and polymeric photonic crystals were characterized using a scanning electron microscope (S-4800, HITACHI, Tokyo, Japan). Reflectance spectra were tested using a fiber spectrometer (AvaSpec-ULS2048, Avantes, Apeldoorn, Netherlands). These spectra were taken with light impinging perpendicular to the sensors. The diameter of the collection spot was about 1.5 mm. The color change of the polymeric photonic crystals was recorded using a common digital camera.

## 3. Results and Discussion

**Figure 1:**
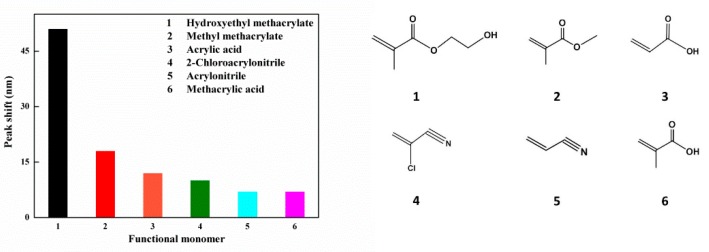
Peak shifts of the inverse opal polymeric photonic crystals (IOPPCs) with different functional monomers (30 mM p-nitrophenol (PNP)): (**1**) hydroxyethyl methacrylate; (**2**) methyl methacrylate; (**3**) acrylic acid; (**4**) 2-chloroacrylonitrile; (**5**) acrylonitrile; (**6**) methacrylic acid.

With different functional monomers, the response performance of prepared sensors is different. It is therefore necessary to select functional monomers with which to fabricate the sensors according to the detection target. Under the same conditions, six kinds of IOPPCs were fabricated using functional monomers such as methacrylic acid, acrylic acid, methyl methacrylate, 2-chloroacrylonitrile, acrylonitrile, and hydroxyethyl methacrylate, respectively, and their response performances were tested in 30 mM PNP aqueous solution. As shown in Figure 1, the Bragg diffraction peak of the sensor with HEMA as a functional monomer red shifted by 51 nm, which was significantly more than that of the sensors with other functional monomers. The reason for this is that the sensor built by HEMA is more sensitive to PNP solution and its volume swelling leads to a red shift of the diffraction peak. Hence, HEMA was selected as the functional monomer with which to fabricate IOPPCs for the detection of PNP.

**Figure 2:**
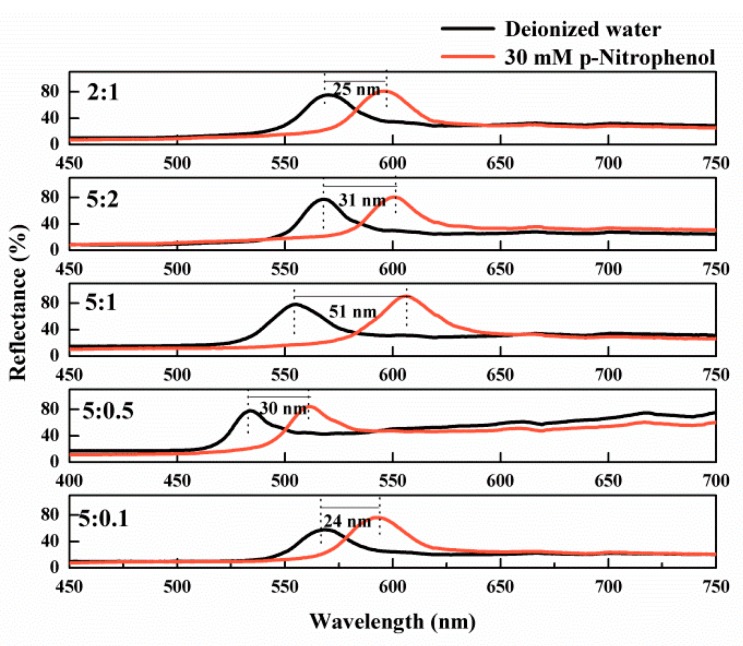
Optical response of IOPPCs fabricated at different ratios of monomer and crosslinker.

To obtain the optimal desired sensors for detecting PNP, the molar ratios of the functional monomer and crosslinker were exploited. Generally, when IOPPCs are fabricated with a lower ratio of crosslinker to functional monomer, they are more sensitive to the PNP [32]. However, when the concentration of the crosslinker is reduced below a certain level, not only is it difficult for the IOPPCs to maintain their 3D-ordered structure but also their flexibility is reduced. Thus, the proportions of functional monomer and crosslinker need to be balanced to obtain the most outstanding sensors [33]. Figure 2 shows IOPPCs fabricated with different ratios of HEMA and EGDMA responding to 30 mM PNP. It can be seen that the Bragg diffraction peak red shifted by 25, 31, 51, 30 and 24 nm when the molar ratios between the monomer and crosslinker were 2:1, 5:2, 5:1, 5:0.5, and 5:0.1, respectively. To this end, a 5:1 molar ratio of monomer to crosslinker was utilized when detecting PNP in this work.

**Figure 3:**
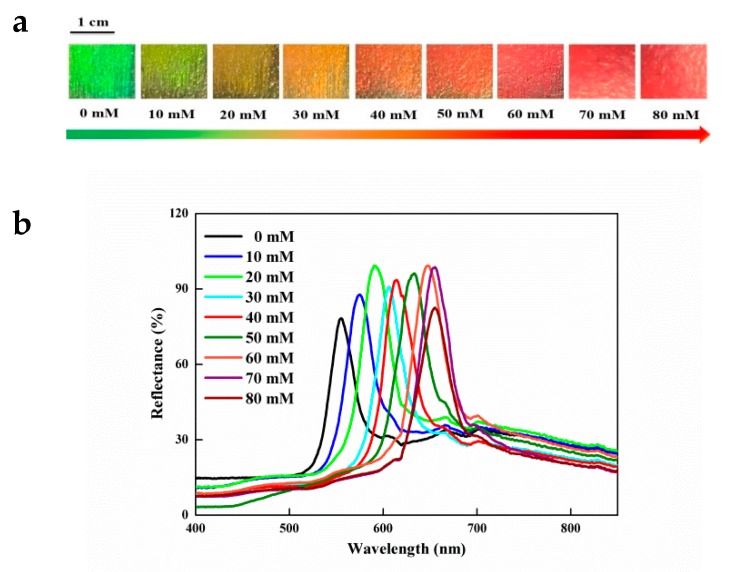
(**a**) Color change of the IOPPCs in different concentrations of PNP; (**b**) optical response of the IOPPCs in different concentrations of PNP.

The sensing behavior of IOPPCs responding to PNP concentrations from 10 to 80 mM is shown in Figure 3. The original Bragg diffraction wavelength of the IOPPCs in deionized water was 555 nm; it red shifted to 576, 590, 606, 613, 633, 647, 654, and 654 nm when the concentration of PNP increased to 10, 20, 30, 40, 50, 60, 70, and 80 mM, respectively. When the concentration of PNP increased to 70 mM or higher, the maximum of the diffraction peaks remained at 654 nm without any shift, indicating that the IOPPCs had reached their maximum swelling limit. Meanwhile, the color of the IOPPCs changed from green to red with the peak shift of Bragg diffraction (Figure 3a). Hence, this change could be directly evaluated by the naked eye for semi-quantitative detection of PNP.

**Figure 4:**
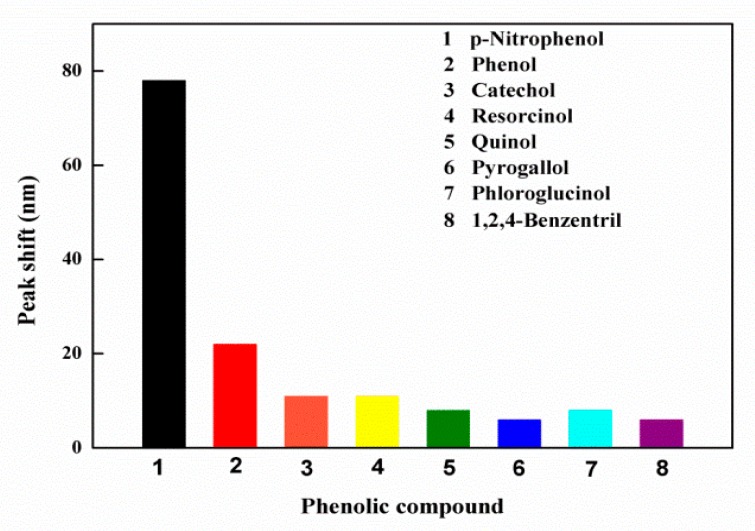
Peak shifts of the IOPPCs in different phenolic compound solutions (50 mM): (**1**) p-nitrophenol; (**2**) phenol; (**3**) catechol; (**4**) resorcinol; (**5**) quinol; (**6**) pyrogallol; (**7**) phloroglucinol; (**8**) 1,2,4-benzentril.

Selectivity is an important factor in evaluating sensors. To demonstrate selectivity to PNP, other kinds of phenolic compounds including phenol, catechol, resorcinol, quinol, pyrogallol, phloroglucinol, and 1,2,4-benzentril, which are structurally similar to PNP, were employed as reference compounds in this study. It was readily apparent that PNP could generate an obvious red shift of up to 78 nm, while other phenolic compounds merely caused a small peak shift (Figure 4). Only PNP could induce a significant shift of the diffraction peak, demonstrating the excellent selectivity of the IOPPCs. Generally, the swelling of polymeric hydrogel depends on the osmotic pressure caused by the free energy of mixing, the hydrogel network elastic restoring force, and the difference in ion concentration between the inside and outside of the hydrogel [17]. Compared with other phenols, because of the electron withdrawing of the nitro group, the molecular polarity and the degree of dissociation in aqueous solution of PNP are different, and its solution is more acidic and its ionic strength higher at the same concentration. IOPPCs with HEMA as the functional monomer have good sensitivity to pH and ionic concentration. In addition, PNP is more likely to interact with the functional groups on the framework of IOPPCs. The swelling of IOPPCs is also influenced by the imbalance of molecular forces in the polymer, such as van der Waals’ force, hydrophobic–hydrophobic attraction, hydrogen bonding, and static interactions [35,36]. Thus, through the selection of functional monomers and the adjustment of crosslinker concentration, the sensors are able to only be sensitive to a specific solution, thus making selective the detection of PNP.

**Figure 5:**
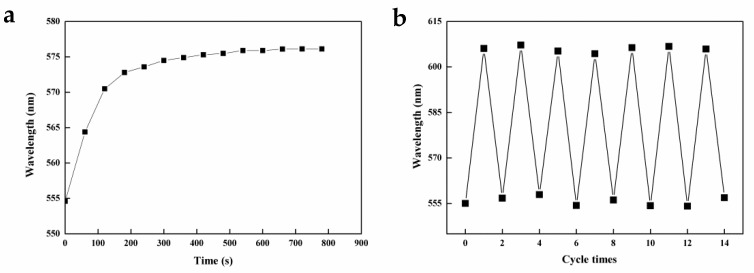
(**a**) Peak shift of the IOPPCs with time (10 mM PNP); (**b**) recoverability of the IOPPCs immersed in PNP solution (30 mM) and recovered by immersion in sodium bicarbonate solution (5%) followed by rinsing with deionized water.

Besides being able to produce a visual optical signal, IOPPCs with 3D-ordered interconnected porous structures have a high surface-to-volume ratio. This is favorable for molecular diffusion, and most of the created functional sites are located at or close to the surface of the ultrathin polymer wall, which contributes to better site accessibility and lower mass-transfer resistance [33]. Generally, the signals were measured when the IOPPCs reached a swelling equilibrium in PNP solution. To observe this equilibrium process, the peak shift of the IOPPCs, which were immersed in 10 mM PNP solution, was recorded. As shown in Figure 5a, the peak shifted fast at the beginning and almost stopped moving after 500 s, meaning that the IOPPCs reached a swelling equilibrium. As IOPPCs are made of highly stable and flexible polymers, they can be easily recovered by immersion in sodium bicarbonate solution (5%) followed by rinsing with deionized water to restore the neutral blank state. As shown in Figure 5b, the standard error was within 5% when the IOPPCs were reused over several cycles in the same PNP solution, indicating the good reproducibility of the IOPPCs. It only took about 20 min to complete the process from the start of detection to restoration of the blank balance.

**Figure 6:**
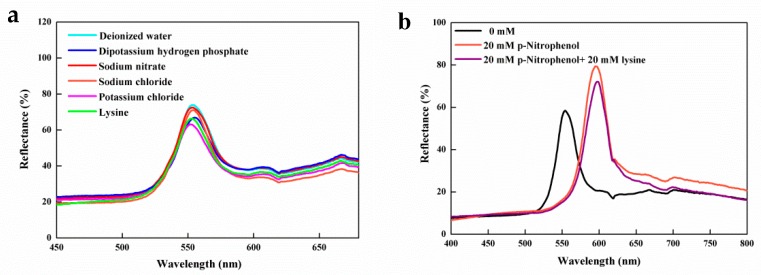
(**a**) Optical response of the IOPPCs in common compound solutions; (**b**) optical response of the IOPPCs in interferents and PNP mixed solution.

The interference immunity for other molecules is also a key factor in the evaluation of IOPPCs. In order to assess the detection ability of the IOPPCs deeply, several common compounds, namely, dipotassium hydrogen phosphate, sodium nitrite, sodium chloride, potassium chloride and lysine (all in 20 mM solution) were chosen to be analyzed by the IOPPCs under the same conditions. As shown in Figure 6a, other compounds almost caused no peak shifts. The reason for this is that IOPPCs are only highly sensitive to a special solution. The molecular structure of these reference compounds and the physicochemical properties of their aqueous solutions are different from those of PNP. As a consequence, they were not able to cause a response of the IOPPCs. Even when the interferents and PNP were detected together, the influence on the shift of the diffraction peak was very small (Figure 6b). For IOPPCs to have good sensitivity to pH and ionic concentration, high concentrations of H^+^ and OH^−^ should be avoided in detection in case of interference.

**Figure 7:**
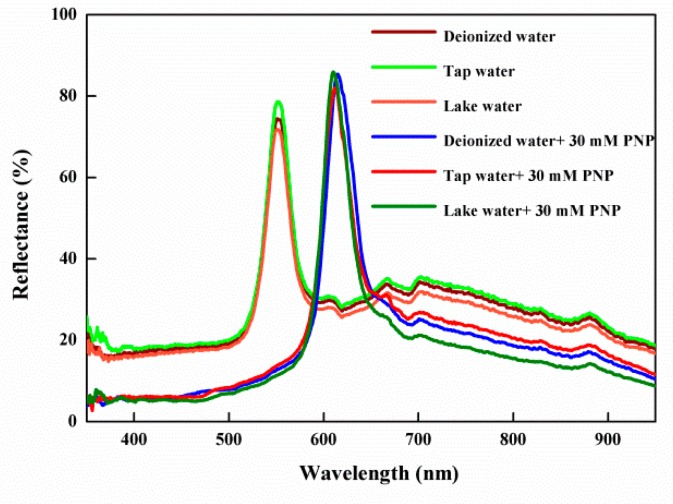
Optical response of the IOPPCs in tap water and lake water.

The results above indicate that IOPPCs have good selectivity and interference immunity. In real environments multiple unknown interference factors could exist. In order to evaluate the applicability of the IOPPCs, tap water and lake water were chosen to be analyzed by the IOPPCs under the same conditions. As shown in Figure 7, there were almost no peak shifts when the IOPPCs were immersed in tap water and lake water, meaning that no PNP (or low concentrations of PNP) had been detected in these samples. After adding 30 mM PNP, the peak shift of the IOPPCs was almost the same as that obtained when measured in deionized water, indicating good applicability.

## 4. Conclusions

In summary, a selective and colorimetric PNP sensor was successfully designed and fabricated based on IOPPCs in this work. The color of the IOPPCs changed from green to red when the concentration of PNP changed from 10 to 70 mM under ambient conditions. Hence, the change can be directly evaluated by the naked eye for semi-quantitative detection of PNP. In addition, the sensor has good stability and satisfying reusability, and it also shows excellent interference immunity. This work will provide a new perspective on the design of sensors for the detection of PNP.

## Supplementary Materials

The following are available online at https://www.mdpi.com/2073-4360/12/1/83/s1, Figure S1: SEM image of the SiO_2_ particles; Figure S2: SEM image of the IOPPCs; Figure S3: Schematic illustrations of the preparation of the IOPPCs.
