# Supplementary material for: Selective and Colorimetric Detection of p-Nitrophenol Based on Inverse Opal Polymeric Photonic Crystals
Source: Polymers (Basel). 2020 Jan 3;12(1):83. doi: 10.3390/polym12010083 (PMC7023634; doi:10.3390/polym12010083)
Supplement: Supplementary file 1 [file polymers-12-00083-s001.pdf]

Supplementary Materials

Selective and Colorimetric Detection of p-Nitrophenol Based on Inverse Opal Polymeric Photonic Crystals

Lu Li [1,2,*], Tiantian Meng [1,2], Wanbin Zhang [1,2], Ying Su [1], Juan Wei [3,*], Xinwei Shi [4] and Guanghua Zhang [1]

1 Key Laboratory of Auxiliary Chemistry and Technology for Chemical Industry, Ministry of Education, Shaanxi University of Science and Technology, Xi'an 710021, China
2 The New Style Think Tank of Shaanxi Universities (Research Center for Auxiliary Chemistry and New Materials Development), Shaanxi University of Science and Technology, Xi' an 710021, China
3 Department of Chemistry, National University of Singapore, 3 Science Drive 3, Singapore 117543, Singapore
4 Engineering Center of QinLing Mountains Natural Products, Shaanxi Academy of Sciences, Xi'an Botanical Garden of Shaanxi Province (Institute of Botany of Shaanxi Province), Xi'an 710061, China
* Correspondence: lilu@sust.edu.cn (L.L.); weijuan@u.nus.edu (J.W.); Tel.: +86-29-8616-8073 (L.L.); +65-6516-8142 (J.W.)

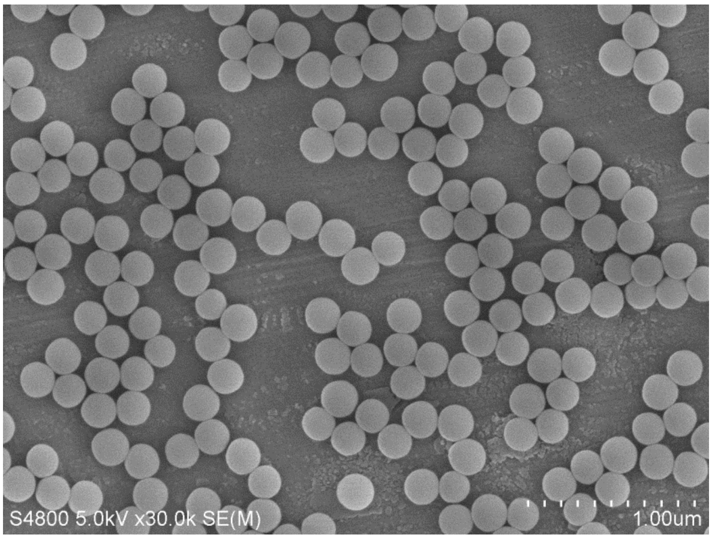

Figure S1. SEM image of the SiO_2 particles

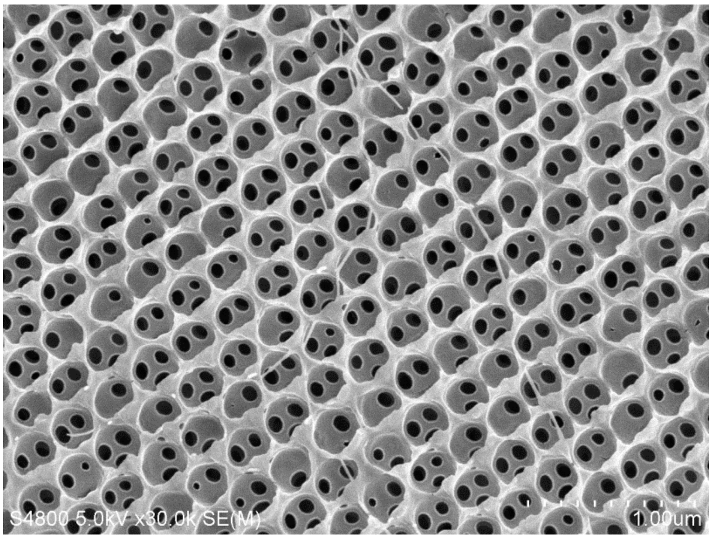

Figure S2. SEM image of the IOPPCs

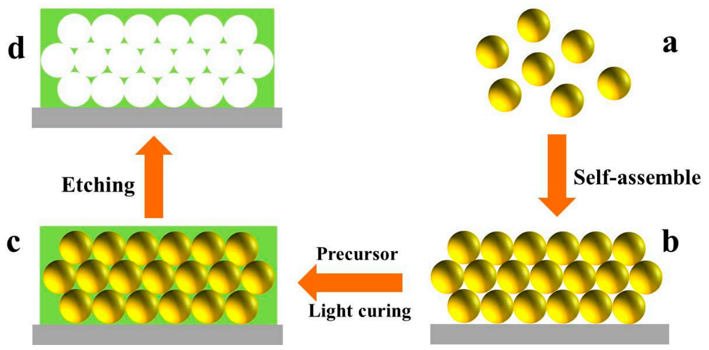

Figure S3. Schematic illustrations of the preparation of the IOPPCs: (**a**) SiO_2 particles; (**b**) photonic crystal templates; (**c**) photonic crystal templates after polymerization; (**d**) inverse opal polymeric photonic crystals.
